# Organised Charity Abroad

**Published:** 1898-12-31

**Authors:** 


### The Institutional Workshop.

### ORGANISED CHARITY ABROAD.

### THE HOUSE OF THE BON SAUVEUR, AT CAEN.

Old Norman chroniclers tell us that William the Conqueror, threatened with excommunication by the Pope for his marriage with Mathilde, bought himself off by consenting to build five hospitals and two monasteries. The hospitals were founded at Rouen, Caen, Bayeux, and Cherbourg. The monasteries, St. Stephen, the Abbaye-aux-Hommes, and La Trinité, the Abbaye-aux-Dames, are still the glory of Caen.

In strange contrast to these foundations, regal alike in traditions as in their size, form, and revenues, stands the institution known as the Bon Sauveur. Insignificant in origin, it faces and meets the needs of the present. We are neither awestruck nor dazzled as we enter its doors; we meet men and women, and we are content to be human.

This institution, so entirely ignored by Englishmen, is well known on the Continent. Its royal letters patent date from 1734. From a civil standpoint its importance arises from its practical usefulness in taking charge of the mentally afflicted of all ages and of both sexes in every phase of their disorder, as also of deaf-mutes. It forms an organised assemblage spreading over many acres of dwelling-houses, courts, quadrangles, workshops, alleys, and gardens—a town within a town. As one enters from the street and advances into the interior of the community grounds, one is struck above all by a certain spirit of practical, honest cheerfulness—the essence and outcome of the life of the place.

The work of organisation rests with the 300 Sisters, who are guided in their gigantic undertaking by liberal, kind-hearted, cultivated, university men, such as were the late Pierre François Jamet and Jean François Desprez, for 40 years proviseur of the Lycée Malherbe at Caen.

The Sisters, as the common proprietors, are responsible to the public for all that takes place within their walls, and it is they who engage or dismiss wardens as immediate attendants on the 600 men confided to them as being in a state of mental aberration. These, with the priests, gardeners, and other workmen employed in the cow-houses, stables, laundry, bakeries, cider-presses, mills, &c., when added to the 900 women patients, and to the several schools under the Sisters' rule, form a motley yet interesting study.

Applications for admission into the institution must contain (1) a certificate of birth or baptism, (2) a declaration of mental disease, (3) and a confirmation of this declaration by some doctor duly authorised to confirm it. Parents are specially invited to allege cause and progress of the malady, remedies prescribed or tried, as also to describe as far as may be the habits, character, and temperament of the patient.

Three classes of patients are admitted, paying at three different rates. First-class patients pay from 1,200 francs and above (about £48); second-class, from 900 francs (£36); and third-class, 650 francs (£26) a year. These sums represent a great difference in diet. The food served to first-class patients is similar to that found at the table d'hôte of the best hotels, while patients of the third class are, of course, in this respect as in many others, much on a level with the peasantry and artisan class as they are in their own homes. For all alike these sums are payable, either terminally or monthly in advance. Parents can make special arrangements as to tobacco, coffee, chocolate, &c. The family provides clothing and linen; and, as is the custom in French schools, a certain specified outfit is always expected except in cases of indigence. The month in which a patient dies or is withdrawn counts as being due to the institution, which also has the right to retain any personal belongings unclaimed within a year. The buildings are so arranged that no communication between the sexes is possible, but the utmost liberty is allowed. Parents may visit whenever the doctors are able to allow it, but strangers are never permitted to interview any patient unless with a written permit from parent or tutor.

An Englishman is very much struck by the outdoor life led by the patients. Fresh air is regarded as of vital importance, and the hours spent under cover are as limited as possible. Such patients as are not violent are allowed to walk as they like in the gardens, and excursions to a country farm belonging to the Sisters are organised every week. Cabs and other conveyances are provided for patients whose families can afford them these luxuries, and the community stables are often able to boast some very fine horses.

One part of the grounds, known as Saint Michel, is set apart for men patients of the first class, who may go to any expense they please in the scale of living. These have a small house to themselves containing four apartments, and may have their own valets, &c. The choice of the individual servants must, however, be subject to the consent of the Sisters, who are, of course, finally responsible for the maintenance of good order. The rooms provided are clean, commodious, airy and even sumptuous. The gentlemen living in this quarter have a refectory in common, and may take their meals there or in their own house as they please. Special arrangements may be made with the authorities for light and firing. A billiard-room is attached to their quarters, and a "salon de réunion," where musical instruments and games are provided for convalescents and harmless patients. There is also a library, to be used with a doctor's permission; also large bath-rooms.

The above, which applies to men patients, is true, with certain small reservations, of the women. Third class patients sent by the departments are housed with a degree of consideration and comfort which is truly astonishing; the spotless dimity hangings and pillows, the cretonne counterpanes, and freshly-painted floors contrast favourably with many boarding-schools for girls whose parents pay a much larger fee; while the alleys and quadrangle provided as their recreation ground remind one very much of a Place in any Norman town. There are the benches where friends and neighbours like to chat and amuse themselves—the little groups exchanging the last news—solitary matrons and young girls knitting or lace-making, others walking up and down in the shade, and all making the best of life with the true French *savoir vivre*.

The "Dames de Famille" form, of course, a community apart, while, as with the men, such as prefer and can afford it have their separate little establishments.

